# Supplementary material for: Elimination of oxygen sensitivity in α-titanium by substitutional alloying with Al
Source: Nat Commun. 2021 Oct 25;12:6158. doi: 10.1038/s41467-021-26374-w (PMC8546145; doi:10.1038/s41467-021-26374-w)
Supplement: Supplementary file 1 — Supplementary Information [file 41467_2021_26374_MOESM1_ESM.docx]

Supplementary Information for:

Elimination of oxygen sensitivity in *α*-titanium by substitutional alloying with Al

**Authors:** Yan Chong^1,2,†^, Ruopeng Zhang^1,2,†^, Mohammad S. Hooshmand^1^, Shiteng Zhao^1,2^, Daryl C. Chrzan^1,3^, Mark Asta^1,3^, J.W. Morris Jr.^1^& Andrew M. Minor^1,2,3,*^

**Affiliations:**

^1^ *Department of Materials Science and Engineering, University of California, Berkeley, CA, USA.*

^2^ *National Center for Electron Microscopy, Molecular Foundry, Lawrence Berkeley National Laboratory, Berkeley, CA, USA.*

^3^ *Materials Sciences Division, Lawrence Berkeley National Laboratory, Berkeley, CA, USA*

† *These authors contributed equally to this work.*

*Correspondence to: [aminor@berkeley.edu](mailto:aminor@berkeley.edu)

**Supplementary Discussion**

**(1) Kernel Average Misorientation (KAM) map analysis of tensile fractured Ti-Al-O**

We characterized the areas near the fracture surfaces (at 77 K) of Ti-Al-O samples. The step size of EBSD scan was 0.05 µm, and KAM values were calculated considering the first nearest neighbor. The obtained inverse pole figure (IPF) maps and corresponding KAM maps are shown in **Supplementary** **Figure 2**, in which the fractured strain was indicated for each microstructure. The KAM map range was 0~5°.

In the Ti-2Al-0.1O sample, a large number of deformation twins was observed, and the KAM values at the grain boundaries and twin boundaries were generally higher than those inside the grains. This reflected a dominant contribution of deformation twins to the excellent strain-hardening ability of Ti-2Al-0.1O at 77 K. With further increase of Al content in low oxygen Ti-Al-O alloys, the deformation twins were gradually suppressed. The plastic deformation was more localized at the grain boundaries, as reflected by the generally larger KAM values at the grain boundaries in Ti-4Al-0.1O and Ti-6Al-0.1O alloys.

In high oxygen Ti-Al-O alloys, the plastic deformation at grain boundaries remained comparably larger than that inside the grains. However, one clear tendency is that, with increasing Al content, the plastic deformation (reflected by the KAM values) became progressively larger inside the grains. This is particularly evident when comparing the KAM maps of Ti-2Al-0.3O and Ti-6Al-0.3O. The distribution of KAM values clearly became more homogenous in the latter. It is believed that, the more homogeneous distribution of plastic strain in Ti-6Al-0.3O alloy can be beneficial for the ductility in two ways. Firstly, the substantial dislocation interactions/cross-slips can provide sufficient strain-hardening ability to sustain the high stress level at 77 K. Secondly, the strain localization at the grain boundaries can be somehow relaxed, preventing/delaying the formation of grain boundary micro-cracks.

**(2) DFT calculations on the interaction between substitutional Al and interstitial O**

**Supplementary** **Figure 4** presents results of DFT calculations of the interaction energy between an Al substitutional solute and interstitial oxygen in both octahedral and hexahedral positions. For the octahedral interstitials the results are largely consistent with those in Ref. [21]. Specifically, our results indicate that, relative to the lowest energy position of the oxygen atom in an octahedral position far from the Al solute, there is a repulsive interaction energy for the nearest neighbor site of 0.71 eV, which is reduced to 0.03 eV at the second neighbor octahedral site. By comparison, the interaction of Al and oxygen in the nearest neighbor hexahedral site shows the highest repulsive value of all sites considered, with a magnitude of 1.60 eV. The interaction energy for the second neighbor hexahedral site is 1.46 eV, while the energy difference between octahedral and hexahedral is 1.10 eV in bulk Ti from the present calculations. We interpret these results to have the following consequences for the experimental results obtained in the present work. Prior to deformation, we expect that oxygen atoms would be positioned at second neighbor octahedral sites to Al or further, due to the large positive interaction energy that is much higher than the thermal energy (kT) for relevant heat treatment temperatures. Thus, if a shuffle of oxygen brings it to a nearest neighbor hexahedral position the energy of the system would be raised by 1.57 eV, compared to a value of 1.10 eV in pure Ti. This should impede the shuffle to a nearest-neighbor site of Al (and make recovery back to the second-neighbor site easier) relative to the situation for bulk Ti. We note also that if oxygen resides in a short-range-ordered domain with a structure similar to the *α*_2_-Ti_3_Al ordered phase, oxygen would be expected to sit in the octahedral sites surrounded by Ti, and shuffle from these sites to any hexahedral neighboring site would result in oxygen residing in a site with a nearest-neighbor Al – based on the results summarized above this scenario would be expected to lead to an increase in energy larger than in bulk Ti.

**(3) Temperature dependences of mechanical properties and dislocation structures**

The mechanical properties of Ti-Al-O alloys also showed dramatically different temperature sensitivities, depending on the oxygen content. The engineering stress-strain curves of low oxygen content (0.1 wt%) alloys **(a)**, and higher oxygen content (0.3 wt%) alloys **(b)** at both room and cryogenic temperatures are shown in **Supplementary** **Figure 5**. The engineering stress-strain curves of pure Ti are also shown in both figures as a reference. In low oxygen content alloys (**Supplementary** **Figure 5(a)**), in addition to the greatly enhanced yield strength, the tensile ductilities at cryogenic temperature were generally comparable to or even better than those at room temperature. The improved tensile ductilities at lower temperature (particularly for Ti-0.1O and Ti-2Al-0.1O alloys) could be attributed to a higher propensity of deformation twinning at cryogenic temperature. In a sharp contrast, the alloys with a higher oxygen content (**Supplementary** **Figure 5(b)**) basically suffered from a loss of tensile ductility with decreasing temperature, especially when the aluminum content was at a low level. Nevertheless, the loss of tensile ductility at lower temperature did gradually diminish with increasing aluminum content.

From a microscopic view, the different temperature sensitivities of mechanical properties in low and higher oxygen content alloys also originated from the different natures of oxygen induced and SRO induced planar slips, as well as the associated *deformation delocalization* abilities. As confirmed in our previous study [11] for the oxygen induced planar slip, there existed a dramatic temperature dependence due to the nature of interstitial shuffling mechanism (ISM), in which an interplay between athermal slip-induced shuffling of oxygen to hexahedral sites and thermal recovery assisted by hopping of oxygen back to octahedral positions determined whether planar slip occurred or not. Following this mechanism, planar slip in high oxygen content alloys is more prone to occur at cryogenic temperature than at room temperature, which has been reflected in the typical dislocation structures in Ti-2Al-0.3O alloy deformed at these two conditions (**Supplementary** **Figure 6(a)**). Considering the poor delocalization ability associated with oxygen induced planar slip, these higher oxygen content alloys typically exhibited a rather limited ductility at cryogenic temperature. On the other hand, in alloys with low oxygen content, where the ISM model was minor and the SRO effect became dominant, i.e. Ti-6Al-0.1O alloy, both the dislocation structures and deformation delocalization abilities were quite similar at both room and cryogenic temperatures (**Supplementary** **Figure 6(b)**). This truly reflected a temperature insensitivity of the deformation behaviors in alloys with SRO, which also explained the unaffected tensile ductility in these alloys at both deformation temperatures.

Finally, it is interesting to see in alloys (i.e. Ti-6Al-0.3O alloy) where both the two mechanisms were possible; the material also exhibited a temperature insensitivity, from the perspectives of both mechanical properties and dislocation structures (**Supplementary** **Figure 6(c)**). This phenomenon also indirectly supported our assumption of suppressed interstitial shuffling mechanism due to the existence of short-range ordered aluminum solutes in a Ti-Al-O ternary system with an aluminum content higher than 6.0 wt%. It is also worth noting that; the twinning activities were basically suppressed in the three alloys at both room and cryogenic temperatures (**Supplementary** **Figure 6**), thereby making the dislocation structure as the dominant factor in affecting the mechanical properties of the three alloys.

**(4) Two-stage mechanism for improved tensile ductility from Ti-0.3O to Ti-6Al-0.3O**

In our previous study of Ti-O alloys [11], we reported for the first time an unusual {11-24} twin in Ti-0.3O alloy at cryogenic temperature deformation. The predominance of this type of twin over other conventional twins was considered as an additional factor leading to the premature fracture of the material at cryogenic temperature deformation, due to the fact that {11-24} twins were prone to initiate micro-cracks at grain boundaries where their twin growth was blocked. However, by simply adding 2.0 wt% aluminum, this type of unusual twin was largely suppressed in Ti-2Al-0.3O alloy at cryogenic temperature, which was believed to be the main reason for the slightly improved tensile ductility as compared with Ti-0.3O alloy. Whereas the dislocation activities in both alloys were characterized by a typical intensive planar slip bands, thereby contributing little to the improved tensile ductility. With a further increase of aluminum content (from 2.0 wt% to 6.0 wt%), all types of twins remained suppressed, while a remarkable increase of the tensile ductility (from 7.0% to 24.0%) was achieved. This phenomenon though, can be attributed to the enhanced dislocation cross-slip and deformation delocalization abilities associated with the higher aluminum content where SRO effect dominated over the mechanical shuffling effect of interstitial oxygen, which has been explained in detail in the main text. Therefore, we can summarize a two-stage mechanism that lying behind the continuously improved tensile ductility of Ti-*x*Al-0.3O alloys (*x*=0, 2, 4 and 6), in which the suppression of detrimental {11-24} twins at 2.0 wt% aluminum was followed by a substantial enhancement in dislocation cross-slip abilities at higher aluminum contents. This two-stage mechanism also shed lights on the development of oxygen-tolerant titanium alloys with further improved mechanical properties.

**Supplementary Figures**


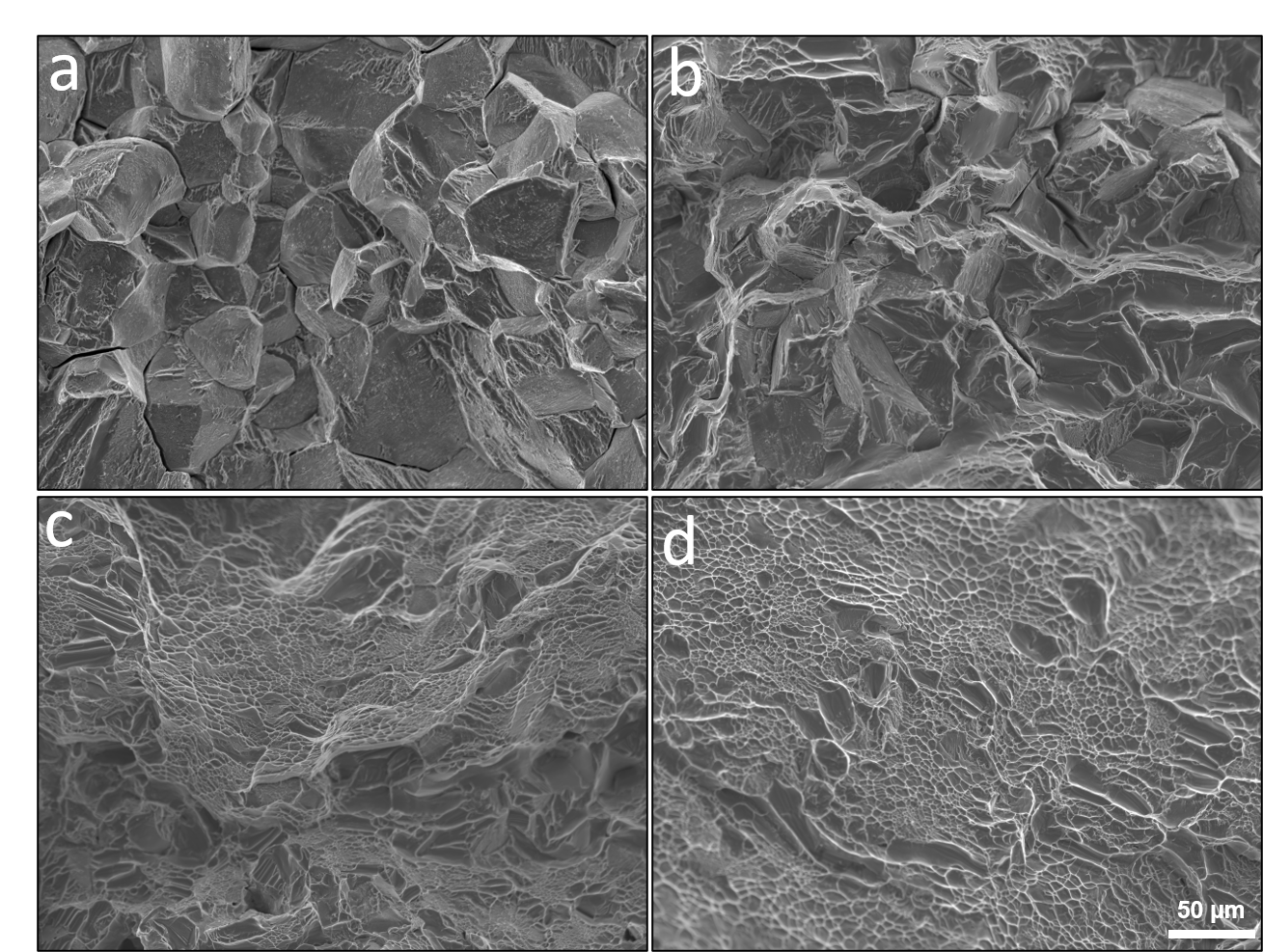


**Supplementary Figure 1**. Typical fracture tomography of Ti-0.3O (**a)**, Ti-2Al-0.3O **(b)**, Ti-4Al-0.3O **(c)** and Ti-6Al-0.3O **(d)** alloys at cryogenic temperature. A gradual transition from intergranular brittle fracture to transgranular ductile fracture was noticed with increasing aluminum content.


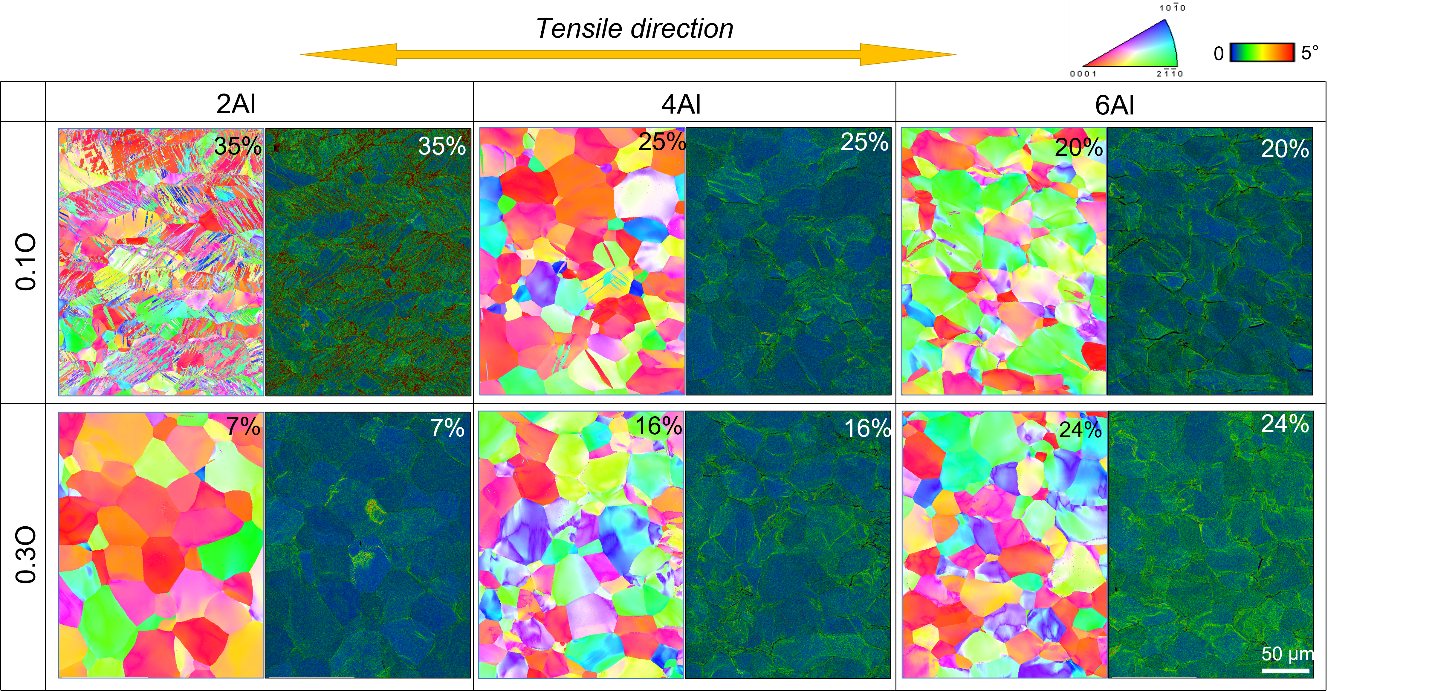


**Supplementary Figure 2.** Inverse pole figure (IPF) maps and corresponding kernel average misorientation (KAM) maps of Ti-Al-O alloy after tensile fractured at 77 K. The range of KAM values is 0-5° for all microstructures. The fracture strain is indicated for each microstructure. Tensile direction is horizontal for all microstructures.


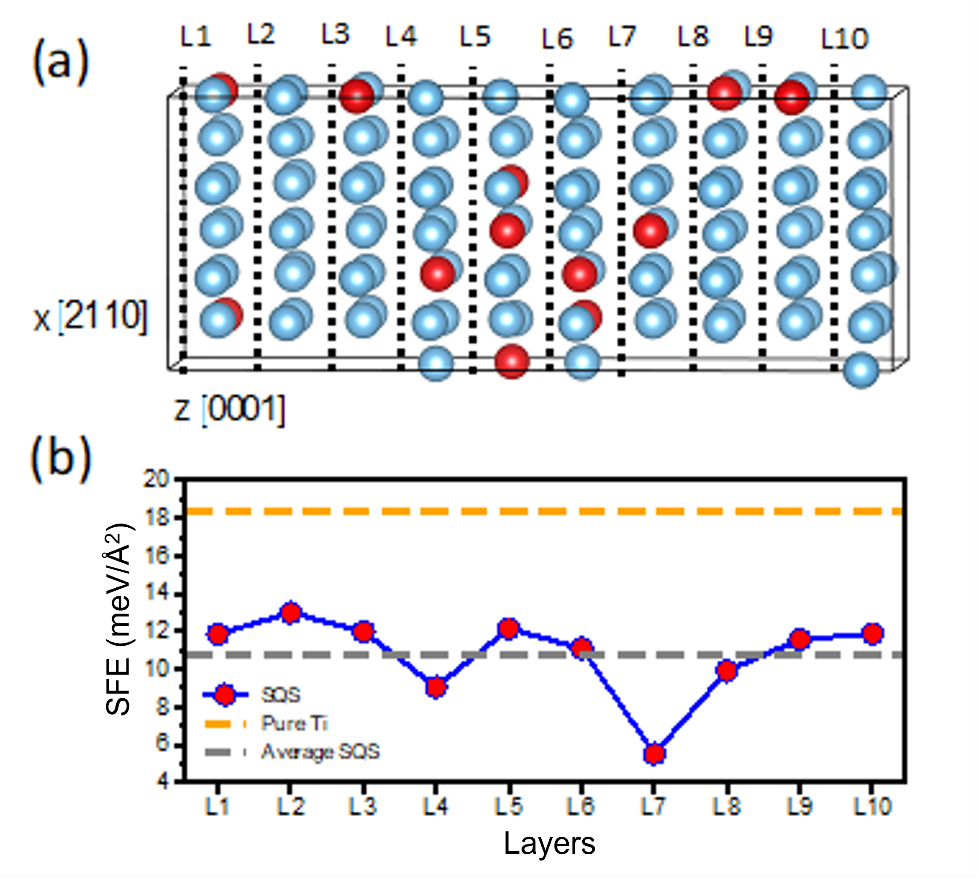


**Supplementary Figure 3.** DFT calculations of Al effect on the stacking fault energy of Ti-Al system. **(a)** Ti-10at.%Al SQS cell with 10 distinct basal planes (labeled L1 to L10) where stacking faults are inserted. Blue and red colors correspond to Ti and Al atoms, respectively. **(b)** Calculated I_2_ SFE of each of the basal planes. Grey and orange lines show the average SFE over all layers in the SQS cell and the I_2_ SFE in pure Ti, respectively.


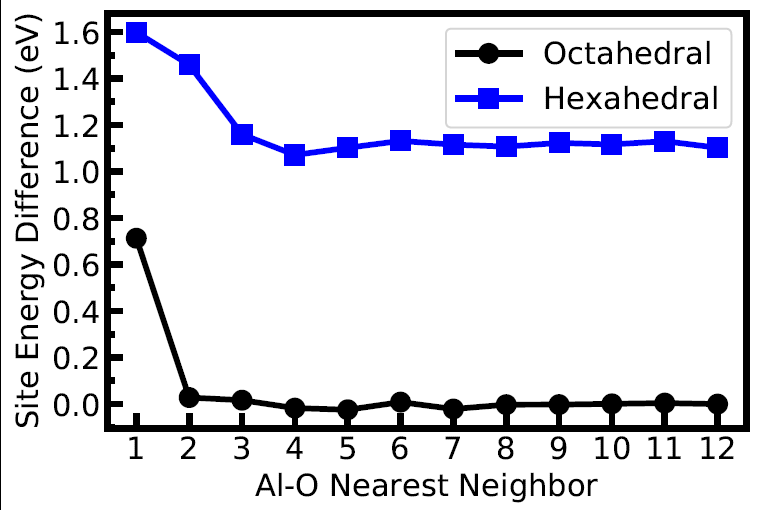


**Supplementary Figure 4.** DFT calculations of the interaction energy between an isolated Al solute atom and oxygen at different neighboring positions in both the octahedral site (black circles) and hexahedral site (blue squares).


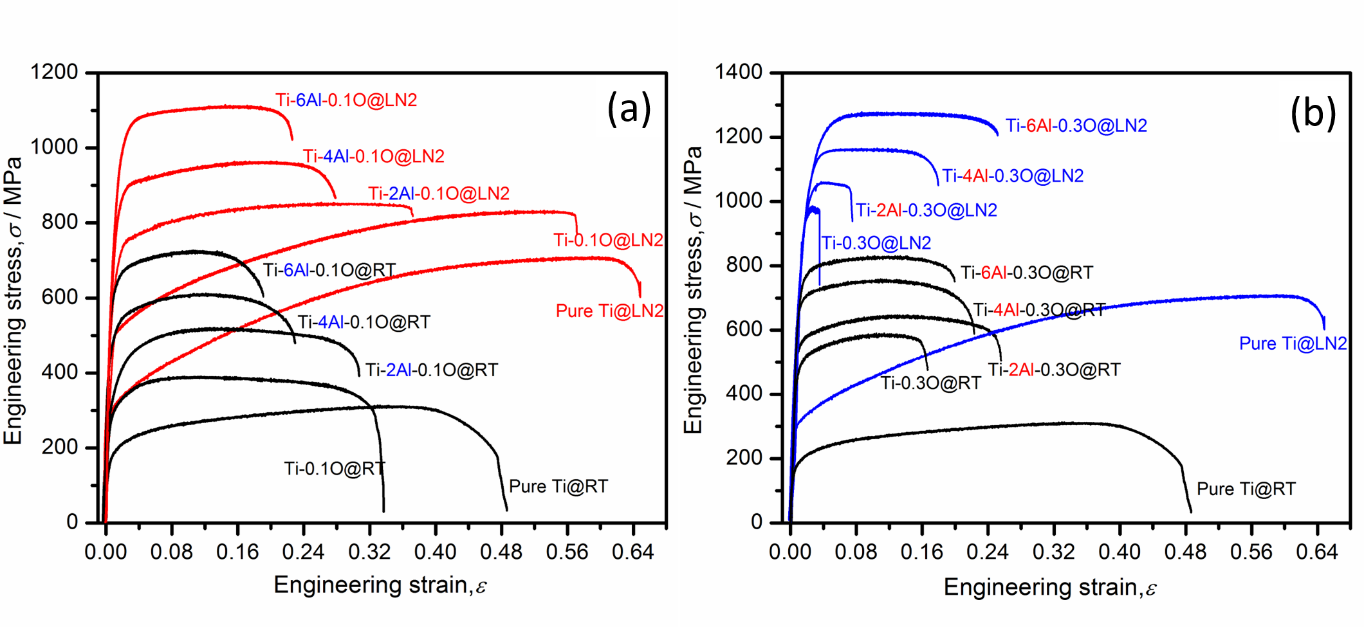


**Supplementary Figure 5.** Engineering stress-strain curves of Ti-*x*Al-0.1O **(a)** and Ti-*x*Al-0.3O **(b)** alloys (*x*=0, 2, 4 and 6) at both room temperature and cryogenic temperature. The engineering stress-strain curves of pure Ti at both temperatures are also shown for reference. In low oxygen system (Ti-*x*Al-0.1O), the temperature sensitivity of tensile ductility was small. In high oxygen system (Ti-*x*Al-0.3O), an acute temperature sensitivity of tensile ductility was found, which, however, was gradually mitigated with increasing aluminum content.


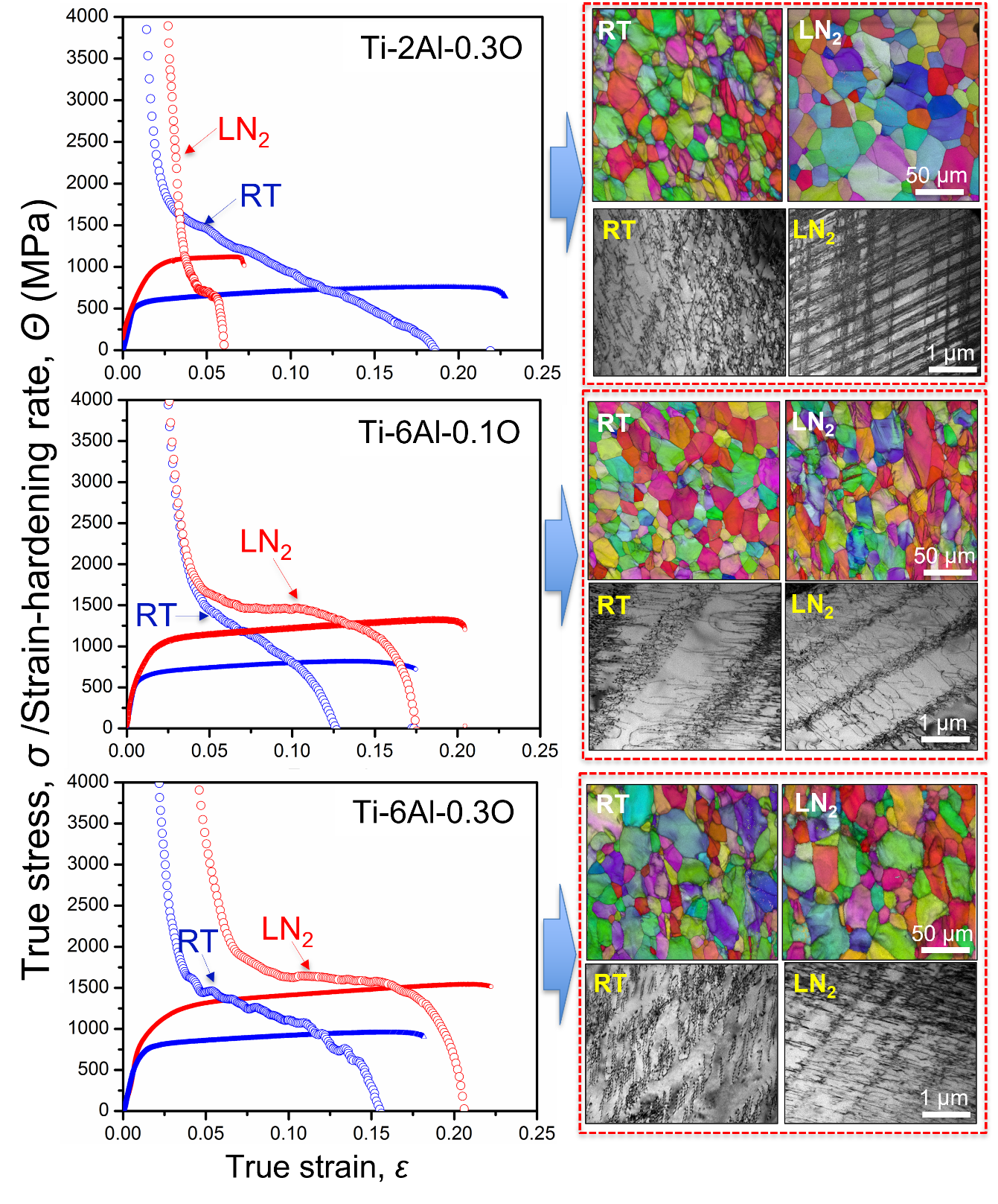


**Supplementary Figure 6.** Temperature dependence (RT and LN_2_) of the mechanical properties and deformation behaviors in Ti-2Al-0.3O, Ti-6Al-0.1O and Ti-6Al-0.3O alloys. The true stress-strain curves and strain-hardening rate curves of the three alloys at both RT and LN_2_ are compared in the first row. EBSD IPF+IQ maps of the tensile fractured samples are shown in the second row. Typical dislocation morphologies of samples tensile deformed by a plastic strain of ~6.0% are shown in the third row.
